# Erector spinae plane block for pain management in lumbar percutaneous vertebroplasty: study protocol for a randomized controlled trial

**DOI:** 10.7717/peerj.20660

**Published:** 2026-01-14

**Authors:** Ali Li, Yun Zhao, Yaru Li, Xuan Wang

**Affiliations:** Department of Anesthesiology, the First Affiliated Hospital of Henan University of Chinese Medicine, Zhengzhou, Henan, China

**Keywords:** Erector spinae plane block, Percutaneous vertebroplasty, Local anesthesia, Pain management, Visual analogue scale

## Abstract

**Background:**

Percutaneous vertebroplasty (PVP) often causes moderate-to-severe intraoperative pain, with current local anesthesia methods providing limited relief. The erector spinae plane block (ESPB) has shown promise in regional analgesia but lacks evidence for PVP. This study aims to test the hypothesis that a preoperative bilateral ESPB provides superior intraoperative analgesia compared to local anesthetic infiltration alone in patients undergoing lumbar PVP. The primary objective is to compare the incidence of moderate-to-severe pain between the two groups.

**Methods:**

This single-center, randomized, single-blind, parallel-group trial will enroll 66 patients undergoing lumbar PVP, who will be randomized to ESPB (20 mL 0.25% ropivacaine bilaterally) or local anesthesia (1% lidocaine). Primary outcome: incidence of moderate-to-severe pain (VAS > 3). Secondary outcomes will include maximum pain scores, patient/surgeon satisfaction, rescue analgesia, time to first mobilization, incidence of adverse events, anesthesia preparation time, and total operating-room occupancy time.

**Discussion:**

The study aims to validate ESPB’s efficacy in PVP, potentially reducing opioid use and improving recovery. Strengths include CONSORT adherence and pragmatic outcomes, though single-center design and limited follow-up may affect generalizability.

**Conclusion:**

This trial will determine whether ESPB offers superior analgesia for PVP, guiding future pain management strategies. If proven effective, ESPB could be adopted as a key component of multimodal analgesia for vertebral augmentation, potentially leading to reduced opioid dependence, improved patient comfort, and faster recovery. The results will guide future evidence-based pain management strategies for these common procedures.

## Introduction

Percutaneous vertebroplasty (PVP) is a minimally invasive surgical procedure widely utilized in the treatment of osteoporotic vertebral compression fractures and vertebral tumors (Haibier et al., 2023). While offering advantages such as minimal invasiveness and rapid recovery, moderate-to-severe pain is frequently experienced by patients during the procedure, often attributed to cement injection and vertebral body expansion (Liu et al., 2024; Della Puppa, Andreula & Frass, 2008). Currently, local anesthesia, such as lidocaine infiltration, is commonly employed during PVP; however, its analgesic efficacy is often limited and may not adequately cover the pain induced by surgical stimulation, particularly the intense pain associated with cement injection (Liu et al., 2024; Della Puppa, Andreula & Frass, 2008). Consequently, the optimization of intraoperative analgesia strategies to enhance patient comfort represents a critical clinical challenge.

In recent years, regional anesthesia techniques have gained increasing prominence in perioperative pain management, with the erector spinae plane block (ESPB) attracting attention due to its ease of performance, favorable safety profile, and effective coverage of the trunk’s nerve supply (Kot et al., 2019; Oostvogels et al., 2024). ESPB involves the injection of local anesthetic either deep or superficial to the erector spinae muscle, leading to the blockade of the dorsal rami and a portion of the ventral rami of spinal nerves, thereby achieving analgesia in the thoracolumbar region (Viderman, Aubakirova & Abdildin, 2022; Liu et al., 2023). Although the analgesic effectiveness of ESPB has been validated in several trials for lumbar spinal fusion, research on its application in PVP remains relatively scarce (Lu et al., 2025; Sharma et al., 2024; Zhang et al., 2021).

Based on its documented mechanism of action, we hypothesize that ESPB can provide effective analgesia for PVP. The cranio-caudal spread of local anesthetic within the fascial plane is known to block the dorsal rami of spinal nerves (which innervate the vertebral periosteum and posterior ligaments) and may also affect the ventral rami *via* paravertebral space diffusion (Coppens, Eochagain & Dewinter, 2023; Bagnoli et al., 2024; Schwartzmann et al., 2018). It is thus postulated that this broader neural blockade can cover the primary nociceptive sources activated during PVP, such as pedicle needle insertion and vertebral body distension.

The present study aims to investigate the analgesic efficacy of ESPB in lumbar PVP, with further analysis of its safety and feasibility. It is anticipated that the findings of this study will offer a novel and optimized approach to anesthetic management in lumbar PVP, ultimately improving the patient’s surgical experience.

## Methods

### Study objective

Explore the analgesic effect of ESPB in lumbar PVP.

### Study design

Single-center, randomized, single-blind, parallel-group, following the CONSORT guidelines and the Declaration of Helsinki.

### Ethical considerations

This study has been approved by the Medical Ethics Committee of the First Affiliated Hospital of Henan University of Chinese Medicine (Ethics Approval Number: 2025HL-446-01), and has been registered in the Chinese Clinical Trial Registry (Registration Number: ChiCTR2500107309). The study protocol followes the CONSORT guidelines and the Declaration of Helsinki. All subjects will sign written informed consent and will have the right to withdraw from the study at any time. All collected data will be anonymized.

### Participants

Inclusion criteria are: Age 18 years or older, American Society of Anesthesiologists (ASA) physical status classification of 2 or 3, elective lumbar PVP involving a single vertebral level.

Exclusion criteria are: Pregnancy, allergy to local anesthetics, coagulopathy, PVP performed under general anesthesia, preoperative use of analgesic medications, history of chronic pain, psychiatric or neurological disorders, refusal to participate in the study.

### Recruitment

**Figure 1:**
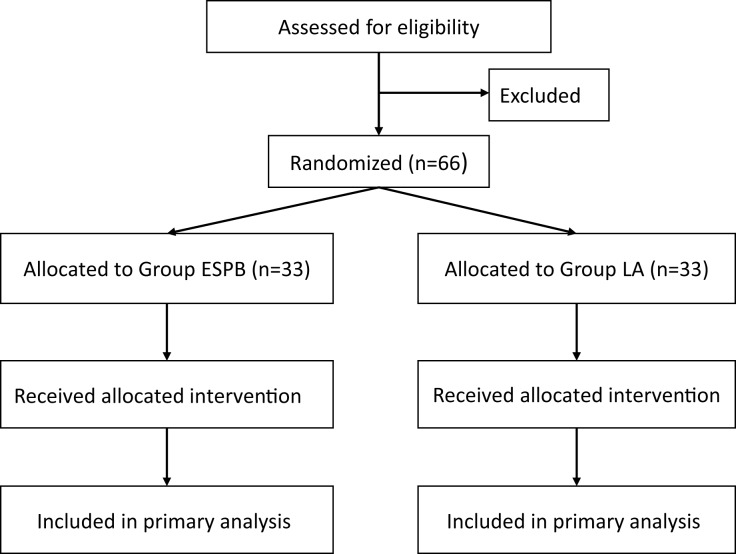
CONSORT flow diagram.

Prospective subjects will be recruited from the Department of Orthopedics at the First Affiliated Hospital of Henan University of Chinese Medicine. All patients scheduled to undergo single-segment PVP under local anesthesia will be screened for eligibility. Eligible patients will be provided with a detailed explanation of the research protocol, including potential benefits and risks, and all questions regarding the study will be addressed. Written informed consent will be obtained from all patients prior to enrollment. It is anticipated that participant recruitment will commence in September 2025. Based on the current surgical volume at our institution, we anticipate that the recruitment phase will last for approximately 4 months, with the entire study (including follow-up and data analysis) expected to be completed by the end of January 2026. The flow of participants through the study will be summarized using a CONSORT diagram, as presented in Fig. 1.

### Randomization

Random numbers will be generated by an independent researcher using the RAND function in Excel, and patients will be divided into local anesthesia group (LA group) and ESPB group in a 1:1 ratio. The random results will be sequentially stored in opaque envelopes, kept by the research coordinator.

### Blinding

Since the LA group requires the surgeon to perform local infiltration anesthesia, it will be impossible to blind the surgeons. Patients, the anesthesiologists responsible for perioperative management, outcome assessors, and statistical analysts will be blinded to group assignment.

A research coordinator will be designated to maintain and distribute randomization numbers and coordinate communication between researchers. After the patient enters the room, the research coordinator will open the envelope and inform an independent anesthesiologist of the patient’s group assignment. This anesthesiologist will leave after the patient’s intervention is completed and will not participate in subsequent research. Another anesthesiologist will be responsible for the patient’s perioperative management. A researcher blinded to group assignment will be responsible for postoperative follow-up.

### Interventions

LA group: Patients will be placed in a prone position. The surgeon will disinfect, drape, and after localization, will use 1% lidocaine for local infiltration anesthesia.

ESPB group: Patients will be placed in a lateral decubitus position. This position is standard for performing a bilateral lumbar ESPB as it provides optimal ergonomics for the operator, facilitates ultrasound probe placement, and allows for stable needle access to the transverse processes on both sides. An independent nerve block specialist (with over 3 years of experience in ultrasound-guided nerve blocks) will perform a bilateral ESPB in the operating room. The block will be performed at the vertebral level corresponding to the planned surgical level (*i.e*., the level of the vertebral fracture). A curvilinear probe (2–5 Hz) will be selected, and a parasagittal approach (approximately 3 cm lateral to the posterior midline) will be used for localization. The transverse process of the target vertebral level will be located, and a 24 G needle will be inserted in-plane to the surface of the transverse process. After negative aspiration for blood and cerebrospinal fluid, 3 mL of normal saline will be injected to separate the erector spinae muscle from the transverse process to confirm correct placement. A total of 20 mL of 0.25% ropivacaine will be administered at this level to achieve cranio-caudal spread of local anesthetic within the fascial plane. The same procedure will be performed on the contralateral side.

Concurrently, during the performance of the nerve block, the surgical team will use this time to prepare the sterile instruments and mix the polymethylmethacrylate bone cement. Upon completion of the bilateral blocks, the patient will be promptly assisted into the prone position. The surgical field will then be disinfected and draped in the standard fashion. A standardized minimum block-to-incision interval of 20 min will be maintained between the completion of the block and the surgical skin incision to allow for adequate sensory blockade onset.

### Perioperative management

No premedication will be administered to any patient preoperatively. Furthermore, to eliminate its potential confounding effect on the primary pain outcome, no intraoperative sedative or anxiolytic medications (*e.g*., midazolam, dexmedetomidine) will be administered to patients in either group. This standardization ensures that the reported pain scores directly reflect the analgesic efficacy of the assigned intervention (ESPB or local anesthesia) without the influence of altered sensorium. Upon arrival in the operating room, an intravenous line will be established in the upper limb, and electrocardiographic monitoring (non-invasive blood pressure, electrocardiogram, pulse oximetry) will be connected. Patients will undergo the appropriate interventions according to their assigned group. If the patient’s visual analogue scale (VAS) pain score is >3 during the operation, sufentanil 5 μg will be injected intravenously, up to a maximum of 15 μg. Flurbiprofen ester 100 mg will be injected intravenously before the end of the surgery. When patients experience moderate-to-severe pain (VAS > 3) in the ward, tramadol 50 mg will be injected intravenously.

### Adverse event monitoring

Adverse events (AEs) will be systematically monitored from the initiation of the anesthesia intervention until patient discharge. AEs of specific interest include local anesthetic systemic toxicity (LAST), pneumothorax, block failure, nerve injury, significant hemorrhage at the block site, and infection.

AEs will be defined according to standard clinical criteria. LAST will be defined as the acute onset of neurological (*e.g*., metallic taste, perioral numbness, seizures, loss of consciousness) or cardiovascular (*e.g*., arrhythmia, hypotension) symptoms following local anesthetic injection (Van Wicklin, 2025). Pneumothorax will be suspected in the presence of acute-onset pleuritic chest pain, dyspnea, oxygen desaturation, or diminished breath sounds on auscultation, and confirmed by chest X-ray (Harnedy, Foley & Fitzgerald, 2025). Block failure will be defined as the inability to achieve a detectable sensory deficit in the surgical dermatomes *via* pinprick test 20 min after ropivacaine injection. All AEs will be assessed and documented by the attending anesthesiologist and the surgical team.

Management protocol: LAST: In the event of suspected LAST, the “Stop, Call, Lipid” protocol will be initiated immediately: (1) Stop injection of local anesthetic; (2) Call for help; (3) Administer a 20% lipid emulsion bolus (1.5 mL/kg) intravenously over 2–3 min, followed by a continuous infusion (0.25 mL/kg/min). Advanced Cardiac Life Support protocols will be followed, with adjustments (*e.g*., reduced epinephrine dose) as per LAST treatment guidelines (Van Wicklin, 2025). Pneumothorax: For a suspected pneumothorax, supplemental oxygen will be administered immediately. A chest X-ray will be obtained for confirmation. A consulting cardiothoracic surgeon will be notified. For a large or symptomatic pneumothorax, needle decompression followed by chest tube insertion will be performed (Harnedy, Foley & Fitzgerald, 2025). Other AEs: Significant hemorrhage or neurological deficits will be promptly investigated with ultrasound or other appropriate imaging (*e.g*., MRI) and managed in consultation with relevant specialists (*e.g*., neurologists, vascular surgeons).

All AEs, regardless of their relation to the study intervention, will be recorded in the Case Report Form (CRF). Serious AEs will be reported to the Principal Investigator and the Medical Ethics Committee within 24 h.

### Data collection

Following the provision of written informed consent by the patients, baseline data will be collected. To standardize the procedural timeline and assess the impact of the interventions on workflow, the following time points will be recorded for all patients: the time of entering the operating room (T1), the time of completion of the nerve block or local anesthesia infiltration (T2), the time of surgical skin incision (T3), and the time of leaving the operating room (T4). The ‘anesthesia preparation time’ will be defined as the interval from entering the operating room (T1) to the completion of anesthesia intervention (T2). The ‘total operating-room occupancy time’ will be defined as the interval from entering (T1) to leaving (T4) the operating room. The ‘block-to-incision’ interval (T2 to T3) will also be calculated. The anesthesiologist responsible for the patient’s perioperative management, who is blinded to group assignment, will assess and record the patient’s VAS pain score at three specific timepoints: (1) during trocar insertion into the pedicle, (2) during polymethylmethacrylate bone cement injection, and (3) at the end of the procedure before transferring the patient out of the operating room. The intraoperative incidence of moderate-to-severe pain will be recorded. Postoperatively, the highest intraoperative pain score, patient satisfaction with intraoperative analgesia, and surgeon satisfaction will be obtained. The incidence of rescue analgesia within the first 24 h post-surgery, as well as the time to first mobilization, will be collected by an independent researcher. The incidence of adverse events will be recorded from the initiation of the intervention until patient discharge.

All data will be initially recorded on paper-based CRF. These data will then be entered into an Excel database, with verification performed by two independent individuals. Although an independent data monitoring committee was not established for this single-center trial, data accuracy and protocol adherence will be safeguarded through internal quality control processes under the supervision of the principal investigator and the institutional ethics committee.

### Measures

#### Primary outcomes

The incidence of moderate-to-severe pain during the operation. This is defined as the proportion of patients reporting a VAS score > 3 at any of the predefined intraoperative timepoints (trocar insertion, cement injection, or end of procedure). Pain will be assessed using a VAS score (0–10), where a higher score indicates more severe pain.

#### Secondary outcomes

Anesthesia preparation time: Defined as the time from patient entry into the operating room to the completion of the assigned anesthesia procedure (ESPB or local infiltration).

Total operating-room occupancy time: Defined as the time from patient entry into to exit from the operating room.

Maximum intraoperative pain score: This will be evaluated using the VAS, ranging from 0 to 10, where higher scores indicated greater pain intensity.

Intraoperative patient satisfaction with analgesia: This will be assessed *via* the VAS, ranging from 0 to 10, with higher scores reflecting greater satisfaction with the pain management provided.

Surgeon satisfaction with analgesia: Surgeon satisfaction will be evaluated using the VAS, ranging from 0 to 10, where higher scores represented greater satisfaction with the analgesia.

Incidence of rescue analgesia within the first 24 h postoperatively: This will be analyzed as a binary outcome (yes/no), representing the proportion of patients requiring any supplemental pain medication within the specified timeframe.

Total perioperative opioid consumption: The total consumption of all rescue opioids administered from the start of the procedure until 24 h postoperatively will be recorded. Intraoperative sufentanil and postoperative tramadol doses will be converted to intravenous morphine milligram equivalents (MME).

Time to first mobilization: This will be defined as the time elapsed until the patient is able to get out of bed for the first time following the procedure.

Incidence of adverse events: Adverse events, including local anesthetic toxicity, hemorrhage, nerve injury or infection, and pneumothorax, will be recorded.

#### Data analysis

Statistical analysis will be performed using SPSS 27.0. The Shapiro-Wilk test will be used to assess normality. Normally distributed quantitative variables will be expressed as mean ± standard deviation, and compared between two groups using an unpaired *t*-test. Non-normally distributed quantitative variables will be expressed as median (interquartile range), and compared between two groups using the Mann-Whitney *U* test. Categorical variables will be expressed as number (percentage), and compared using the chi-square test or Fisher’s exact test. A *p*-value of less than 0.05 will be considered statistically significant.

All analyses will be conducted on an intention-to-treat basis. Any missing data will be handled based on the pattern and amount of missingness. For the primary outcome (incidence of moderate-to-severe pain), a missing value will be considered a treatment failure (*i.e*., included in the numerator for the incidence calculation) to provide a conservative estimate of the intervention’s effect. For secondary continuous outcomes (*e.g*., VAS scores), if data are missing at random and the proportion is small (<5%), complete-case analysis will be employed. If the proportion is larger, multiple imputation methods will be considered.

#### Sample size

According to our center’s data, the incidence of moderate-to-severe pain during lumbar PVP under local anesthesia is 70%. Assuming that ESPB can reduce the incidence of moderate-to-severe pain by 50% (*i.e*., to 35%) (Sharma et al., 2024), with α = 0.05, 1 − β = 0.8, and performing a two-sided test, the sample size was calculated to be 62 cases (31 cases per group) using PASS 2023 software. Assuming a dropout rate of 5%, the final number of cases to be included is 66 (33 cases per group).

## Discussion

The present randomized, single-blind, parallel-group trial is designed to provide the evidence on the effectiveness of ultrasound-guided ESPB for analgesia during lumbar PVP. Although ESPB has proved valuable in lumbar spinal fusion (Lu et al., 2025; Sharma et al., 2024; Zhang et al., 2021), its role in PVP remains largely unexplored. By directly comparing ESPB with routine local infiltration anesthesia, this study seeks to clarify whether a bilateral ESPB can meaningfully reduce the incidence of moderate-to-severe intra-operative pain, improve patient and surgeon satisfaction, and facilitate earlier mobilization without increasing adverse events.

Several design features strengthen the anticipated validity and applicability of the results. First, strict adherence to CONSORT standards, pre-registration and detailed data handling procedures reduce the risk of selective reporting. Second, the sample-size calculation is based on a clinically relevant absolute risk reduction (70% to 35%), ensuring adequate statistical power. Third, clinically pragmatic outcome measures—VAS, rescue-analgesic requirements and time to first mobilization—directly reflect peri-operative care priorities and are easily reproducible in other centers. Finally, performing the block at the surgical vertebral level and using a moderate volume (20 mL of 0.25% ropivacaine per side) mirrors everyday practice (Lu et al., 2025; Sharma et al., 2024; Zhang et al., 2021), increasing external validity. Furthermore, we have implemented a coordinated parallel workflow where surgical preparation occurs simultaneously with the performance of the ESPB, coupled with a mandated minimum block-to-incision interval. This design specifically addresses the potential confounding effect of procedural timing, ensuring that the analgesic efficacy of the ESPB is evaluated after it has reached its peak effect.

Mechanistically, ESPB is expected to exert its analgesic effect through cranio-caudal spread of local anesthetic in the fascial plane superficial to the transverse processes, blocking the dorsal rami implicated in vertebral periosteum innervation and, to a lesser extent, the ventral rami that contribute to nociceptive signaling from paravertebral tissues (Coppens, Eochagain & Dewinter, 2023; Bagnoli et al., 2024; Schwartzmann et al., 2018). Adequate blockade of these pathways may blunt the sharp augmentation of pain commonly observed during cement injection and vertebral body expansion, translating into lower sympathetic activation, greater patient cooperation and shorter procedural times.

Should the trial confirm a meaningful benefit, the findings would support the integration of ESPB into standardized multimodal analgesic protocols for vertebral augmentation procedures. This integration could yield significant downstream advantages, including a structured reduction in peri-operative opioid exposure, smoother workflow for surgeons and anesthesiologists and improved early functional recovery—an outcome of particular relevance in elderly osteoporotic populations where prompt mobilization curtails deconditioning and secondary complications. Conversely, if ESPB confers little or no advantage, future research can redirect resources toward alternative regional techniques or pharmacologic strategies. It is worth noting that other regional techniques, such as the paravertebral block, could also theoretically provide analgesia for PVP. However, the ESPB was selected for this trial due to its perceived favorable safety profile, with a more superficial and posterior injection site potentially reducing the risk of pneumothorax and visceral injury compared to the paravertebral approach, alongside its technical ease of execution (Huang & Liu, 2020; Moustafa et al., 2020).

Furthermore, while the primary focus of this initial study is on intraoperative and immediate postoperative analgesia, the potential impact of effective early pain control on long-term outcomes—such as the development of persistent pain, chronic opioid use, and functional recovery—represents a critical area for future investigation once the short-term efficacy of ESPB is established. Should the short-term results of this trial prove positive, the logical next steps would involve a larger, multicenter validation study and subsequent research with extended follow-up periods to investigate long-term functional outcomes and the potential impact on chronic pain development.

### Limitations

First, conducting the trial at one tertiary hospital may limit generalizability to different surgical teams, patient demographics and institutional protocols. Furthermore, as all ESPB procedures will be performed by a single, experienced operator to ensure consistency, the results may not be fully generalizable to clinical settings where practitioners have varying levels of expertise in ultrasound-guided regional anesthesia. Second, while patients, outcome assessors and data analysts are blinded, surgeons cannot be masked to group allocation, introducing a possibility of performance bias in intra-operative conduct. Third, only ASA II–III adults undergoing single-level lumbar PVP are eligible. Results may not extrapolate to multilevel PVP, thoracic procedures, general-anesthesia cases or patients with severe systemic disease. Fourth, all ESPB procedures in this trial will be performed by a single, experienced nerve block specialist to ensure consistency and technical quality. While this minimizes internal performance variability, it may limit the generalizability of our results to settings where operators have different levels of expertise with ultrasound-guided regional anesthesia. Future implementation of this technique would benefit from standardized training protocols to ensure reproducible efficacy. Fifth, the use of local infiltration as the control intervention, instead of a sham (saline) ESPB, means that our design cannot fully isolate the specific “block effect” from potential non-specific effects or the profound difference in procedural experience between the groups. This was a deliberate choice informed by ethical considerations and our goal to conduct a pragmatic trial comparing two distinct clinical strategies. Finally, outcomes are monitored up to 24 h post-procedure; the study will not capture delayed complications (*e.g*., rebound pain, cement leakage-related irritation) or long-term outcomes such as the development of persistent pain or chronic opioid use.

## Supplemental Information

10.7717/peerj.20660/supp-1 Supplemental Information 1 Protocol.

10.7717/peerj.20660/supp-2 Supplemental Information 2 SPIRIT checklist.

## Additional Information and Declarations

### Data Availability

The following information was supplied regarding data availability:

This is a registered report, stage 1.

### Clinical Trial Registration

The following information was supplied regarding Clinical Trial registration:

ChiCTR2500107309.
