# Supplementary material for: Erector spinae plane block for pain management in lumbar percutaneous vertebroplasty: study protocol for a randomized controlled trial
Source: PeerJ. 2026 Jan 14;14:e20660. doi: 10.7717/peerj.20660 (PMC12811960; doi:10.7717/peerj.20660)
Supplement: Supplemental Information 1 [file peerj-14-20660-s001.doc]

**Erector spinae plane block for pain management in lumbar percutaneous vertebroplasty: a randomized controlled trial**

**Introduction**

Percutaneous vertebroplasty (PVP) is a minimally invasive surgical procedure widely utilized in the treatment of osteoporotic vertebral compression fractures and vertebral tumors [1]. While offering advantages such as minimal invasiveness and rapid recovery, moderate to severe pain is frequently experienced by patients during the procedure, often attributed to cement injection and vertebral body expansion [2, 3]. Currently, local anesthesia, such as lidocaine infiltration, is commonly employed during PVP; however, its analgesic efficacy is often limited and may not adequately cover the pain induced by surgical stimulation, particularly the intense pain associated with cement injection [2, 3]. Consequently, the optimization of intraoperative analgesia strategies to enhance patient comfort represents a critical clinical challenge.

In recent years, regional anesthesia techniques have gained increasing prominence in perioperative pain management, with the erector spinae plane block (ESPB) attracting attention due to its ease of performance, favorable safety profile, and effective coverage of the trunk's nerve supply [4, 5]. ESPB involves the injection of local anesthetic either deep or superficial to the erector spinae muscle, leading to the blockade of the dorsal rami and a portion of the ventral rami of spinal nerves, thereby achieving analgesia in the thoracolumbar region [6]. Although the analgesic effectiveness of ESPB in thoracic and abdominal surgeries has been demonstrated in several studies, research on its application in PVP remains relatively scarce [7-9].

The present study aims to investigate the analgesic efficacy of ESPB in lumbar PVP, with further analysis of its safety and feasibility. It is anticipated that the findings of this study will offer a novel and optimized approach to anesthetic management in lumbar PVP, ultimately improving the patient's surgical experience.

**Study objective**

Explore the analgesic effect of ESPB in lumbar PVP.

**Participants**

Inclusion criteria were: Age ≥ 18 years, American Society of Anesthesiologists (ASA) physical status classification of 2 or 3, elective lumbar PVP involving a single vertebral level.

Exclusion criteria were: Pregnancy, allergy to local anesthetics, coagulopathy, PVP performed under general anesthesia, preoperative use of analgesic medications, history of chronic pain, psychiatric or neurological disorders, refusal to participate in the study.

**Study design**

Single-center, randomized, single-blind, parallel-group.

**Sample size**

According to our center's data, the incidence of moderate-to-severe pain during lumbar PVP under local anesthesia is 70%. Assuming that ESPB can reduce the incidence of moderate-to-severe pain by 50% (i.e., to 35%), with α=0.05, 1-β=0.8, and performing a two-sided test, the sample size was calculated to be 62 cases (31 cases per group) using PASS 2023 software. Assuming a dropout rate of 5%, the final number of cases to be included is 66 (33 cases per group).

**Randomization**

Random numbers were generated by an independent researcher using the RAND function in Excel, and divided into local anesthesia group (LA group) and ESPB group in a 1:1 ratio. The random results were sequentially stored in opaque envelopes, kept by the research coordinator. The blinding key was sealed and kept by the principal investigator.

**Blinding**

Since the LA group requires the surgeon to perform local infiltration anesthesia, it is impossible to blind the surgeons. Patients, the anesthesiologists responsible for perioperative management, outcome assessors, and statistical analysts were all blinded to group assignment.

A research coordinator was designated to maintain and distribute randomization numbers and coordinate communication between researchers. After the patient entered the room, the research coordinator opened the envelope and informed an independent anesthesiologist of the patient's group assignment. This anesthesiologist left after the patient's intervention was completed and did not participate in subsequent research. Another anesthesiologist was responsible for the patient's perioperative management. A researcher blinded to group assignment was responsible for postoperative follow-up.

**Interventions**

LA group: Patients were in a prone position. The surgeon disinfected, draped, and after localization, used 1% lidocaine for local infiltration anesthesia.

ESPB group: Patients were in a lateral decubitus position. An independent nerve block specialist (with over 3 years of experience in ultrasound-guided nerve blocks) performed the ESPB at the surgical segment. A curvilinear probe (2-5 Hz) was selected, and a parasagittal approach (approximately 3 cm lateral to the posterior midline) was used for localization. The transverse process was located, and a 24 G needle was inserted in-plane to the surface of the transverse process. After negative aspiration for blood and cerebrospinal fluid, 3 mL of normal saline was injected to separate the erector spinae muscle from the transverse process to confirm correct placement. 20 mL of 0.25% ropivacaine was administered. The same procedure was performed on the contralateral side.

**Perioperative Management**

No premedication was administered to any patient preoperatively. Upon arrival in the operating room, an intravenous line was established in the upper limb, and electrocardiographic monitoring (non-invasive blood pressure, electrocardiogram, pulse oximetry) was connected. Patients were subjected to the appropriate interventions according to their assigned group. If the patient's visual analogue scale (VAS) pain score was >3 during the operation, sufentanil 5ug was injected intravenously, up to a maximum of 15ug. Flurbiprofen ester 100mg was injected intravenously before the end of the surgery. When patients experienced moderate to severe pain in the ward, tramadol 50mg was injected intravenously.

**Measures**

**Primary outcomes**

The incidence of moderate to severe pain during the operation. Pain was assessed using a VAS score (0-10), where a higher score indicates more severe pain. Moderate to severe pain was defined as a VAS score > 3.

**Secondary outcomes**

Maximum intraoperative pain score: This was evaluated using the VAS, ranging from 0 to 10, where higher scores indicated greater pain intensity.

Intraoperative patient satisfaction with analgesia: This was assessed via the VAS, ranging from 0 to 10, with higher scores reflecting greater satisfaction with the pain management provided.

Surgeon satisfaction with analgesia: Surgeon satisfaction was evaluated using the VAS, ranging from 0 to 10, where higher scores represented greater satisfaction with the analgesia.

Incidence of rescue analgesia within the first 24 hours postoperatively: This refers to the proportion of patients requiring additional pain medication within the specified timeframe.

Time to first mobilization: This was defined as the time elapsed until the patient was able to get out of bed for the first time following the procedure.

Incidence of adverse events: Adverse events, including local anesthetic toxicity, hemorrhage, nerve injury or infection, and pneumothorax, were recorded.

**Data analysis**

Statistical analysis was performed using SPSS 27.0. The Shapiro-Wilk test was used to assess normality. Normally distributed quantitative variables were expressed as mean ± standard deviation, and compared between two groups using an unpaired *t*-test. Non-normally distributed quantitative variables were expressed as median (interquartile range), and compared between two groups using the Mann-Whitney *U* test. Categorical variables were expressed as number (percentage), and compared using the chi-square test or Fisher's exact test. A *p*-value of less than 0.05 was considered statistically significant.

**Ethical requirements and informed consent form**

Clinical trials shall be conducted in accordance with the Declaration of Helsinki and China's relevant regulations on the management of clinical trials. Clinical trials may only be conducted after the trial protocol has been approved by the clinical research ethics committee prior to the commencement of the trial.

Prior to inclusion in this study, the research physician is responsible for providing each patient with a written explanation of the study’s purpose, procedures, and potential risks, ensuring that patients are aware of their right to withdraw from the study at any time. A written informed consent form must be provided to each patient prior to inclusion, and the research physician is responsible for obtaining informed consent from each patient before they enter the study. The informed consent form must be retained as part of the clinical trial documentation for reference. For this trial, both the patient and their authorised representative in an anaesthetised state must be informed of the above content prior to surgery and sign the informed consent form as evidence.

**Organisational Management**

This study is a single-centre study, with Dr. Li Ali from the Department of Anaesthesiology at the First Affiliated Hospital of Henan University of Traditional Chinese Medicine serving as the principal investigator. Their responsibilities include study design, data analysis, and manuscript preparation.

During the study period, monitoring, data entry and verification, and statistical analysis will be conducted by the First Affiliated Hospital of Henan University of Traditional Chinese Medicine.

**Data Retention**

The investigator and sponsor shall properly retain all documents and materials related to the clinical trial in accordance with GCP requirements, with a retention period of five years.

**References**

[1] Haibier A, Yusufu A, Lin H, et al. Effect of different cement distribution in bilateral and unilateral Percutaneous vertebro plasty on the clinical efficacy of vertebral compression fractures. BMC Musculoskelet Disord. 2023;24(1):908.

[2] Liu Y, Wang D, Chi W, Hu F. Study on the combination of remazolam besylate and sufentanil in elderly patients with percutaneous vertebroplasty. Biotechnol Genet Eng Rev. 2024;40(2):1155-1163.

[3] Della Puppa A, Andreula C, Frass M. Assisted sedation: a safe and easy method for pain-free percutaneous vertebroplasty. Minerva Anestesiol. 2008;74(3):57-62.

[4] Kot P, Rodriguez P, Granell M, et al. The erector spinae plane block: a narrative review. Korean J Anesthesiol. 2019;72(3):209-220. doi:10.4097/kja.d.19.00012

[5] Oostvogels L, Weibel S, Meißner M, et al. Erector spinae plane block for postoperative pain. Cochrane Database Syst Rev. 2024;2(2):CD013763.

[6] Viderman D, Aubakirova M, Abdildin YG. Erector Spinae Plane Block in Abdominal Surgery: A Meta-Analysis. Front Med (Lausanne). 2022;9:812531.

[7] Huang X, Wang J, Zhang J, Kang Y, Sandeep B, Yang J. Ultrasound-guided erector spinae plane block improves analgesia after laparoscopic hepatectomy: a randomised controlled trial. Br J Anaesth. 2022;129(3):445-453.

[8] Ciftci B, Ekinci M, Gölboyu BE, et al. High Thoracic Erector Spinae Plane Block for Arthroscopic Shoulder Surgery: A Randomized Prospective Double-Blind Study. Pain Med. 2021;22(4):776-783.

[9] Zhang Q, Wu Y, Ren F, Zhang X, Feng Y. Bilateral ultrasound-guided erector spinae plane block in patients undergoing lumbar spinal fusion: A randomized controlled trial. J Clin Anesth. 2021;68:110090.
